# Cellular schwannoma arising from the gastric wall misdiagnosed as a gastric stromal tumor: A case report

**DOI:** 10.3892/ol.2013.1752

**Published:** 2013-12-11

**Authors:** GUANGYAO WANG, PING CHEN, LIANG ZONG, LEI SHI, WEI ZHAO

**Affiliations:** 1 Department of Gastrointestinal Surgery, Clinical Medical College of Yangzhou University (Subei People's Hospital of Jiangsu), Yangzhou, Jiangsu 225001, P.R. China; 2 Department of Gastrointestinal Surgery, Graduate School of Medicine, University of Tokyo, Tokyo 113-8655, Japan

**Keywords:** gastric neoplasms, schwannoma, diagnosis

## Abstract

Cellular schwannomas have been previously described at almost every anatomic location of the human body, but reports in the gastric wall are rare. The current study presents a rare case of cellular schwannoma originating from the gastric wall. Computed tomography revealed a 5.6×5.3×4.0-cm^3^ solid mass located in the posterior wall of the stomach. Open laparotomy confirmed its mesenchymal origin. Microscopically, the tissue was composed of spindle-shaped and fascicularly-arranged cells, but mitotic figures were rare. Immunohistochemical staining showed that the tumor was negative for cluster of differentiation (CD)117, CD34, smooth muscle actin and desmin, but positive for S-100 and Ki67. The patient presented no evidence of recurrence and metastasis during follow-up. Gastric cellular schwannomas may be diagnosed by clinical characteristics, histological observations and immunohistochemical markers.

## Introduction

Cellular schwannoma is a rare tumor of the peripheral nerves. The tumor consists of cells that are spindle-shaped in a fascicular or interfelted arrangement (Antoni A), with high cell density and lacking the classic schwannoma reticular zone (Antoni B) and structures in palisade arrangement (Verocay bodies). The tumor cells are abundant, with coarse chromatin and deeply stained nuclei. Therefore, the tumor may easily be misdiagnosed as various different types of spindle cell sarcoma. An intraperitoneal cellular schwannoma may also be mistaken as a gastrointestinal stromal tumor (GIST). The current study presents a rare case of a cellular schwannoma arising from the gastric wall. In addition, the clinical characteristics, diagnosis, differential diagnosis and treatment of the cellular schwannoma in the gastric wall were analyzed. Written informed consent was obtained from the patient.

## Case report

**Figure 1:**
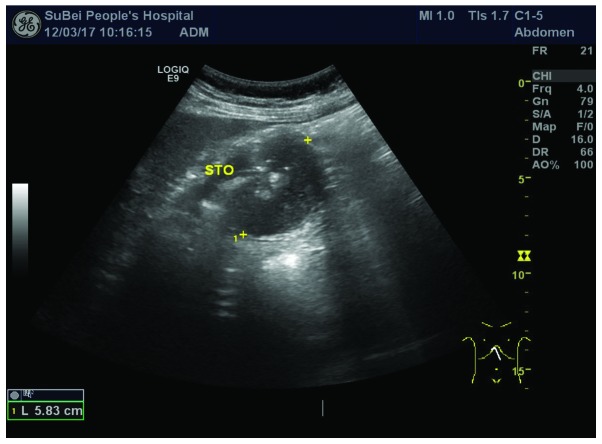
Pre-operative abdominal ultrasound revealing a mass in the posterior wall of the stomach.

**Figure 2:**
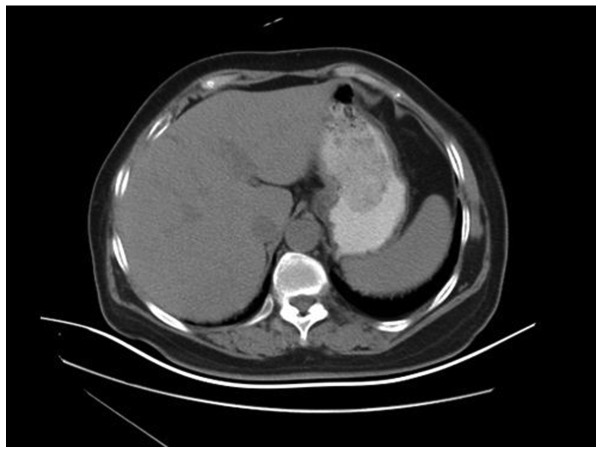
Pre-operative abdominal CT demonstrating a 5.5×5.3-cm solid mass located in the posterior wall of the stomach. Enhanced CT shows peripheral enhancement, low-density areas and a gas shadow. CT, computed tomography.

**Figure 3:**
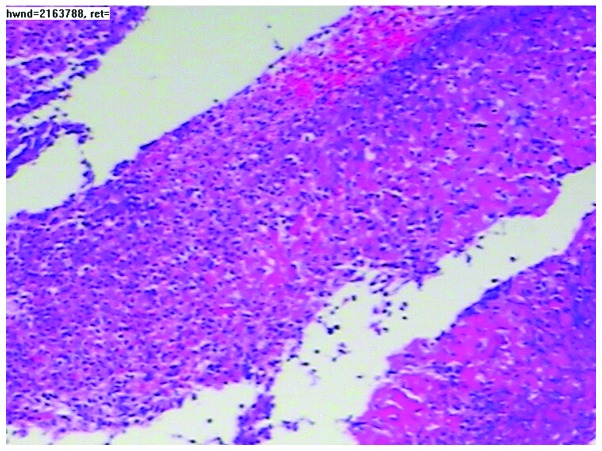
Hematoxylin and eosin-stained pre-operative section. Moderate chronic superficial gastritis was identified, associated with inflammatory exudate and necrotic tissue (magnification, ×100).

**Figure 4:**
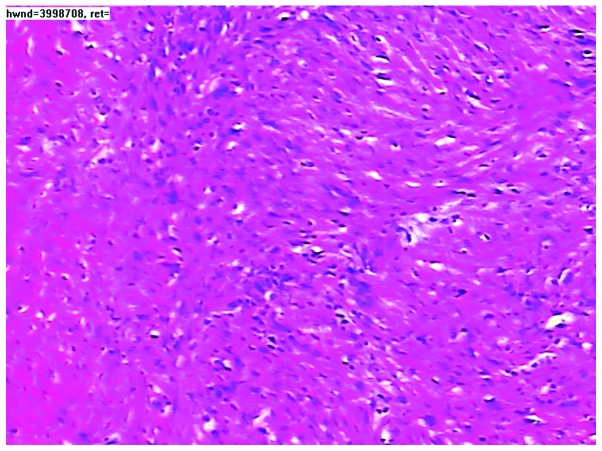
Hematoxylin and eosin-stained post-operative section. Spindle-shaped and fascicularly-arranged cells were identified, but mitotic figures were rare (magnification, ×100).

**Figure 5:**
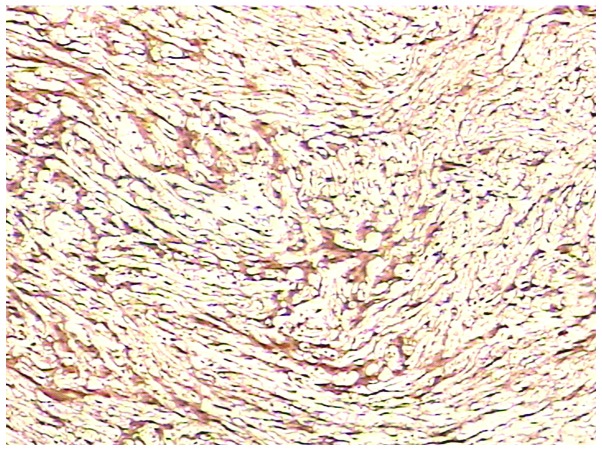
Immunohistochemical test showing that the tumor was positive for S-100 (magnification, ×100).

A 66-year-old female was admitted to the Subei People's Hospital of Jiangsu Province (Yangzhou, China) with the chief complaint of a cervical epidermal cyst, on March 17, 2012. The pre-operative physical examination and laboratory results showed no positive abnormalities, with the exception of a 4×3-cm lump in the right cervical area (Table I). However, pre-operative abdominal ultrasound revealed a mass in the posterior wall of the stomach (Fig. 1). In addition, abdominal contrast-enhanced computed tomography (CT) showed a mass of soft tissue lying behind the wall of stomach (Fig. 2). However, the fiberoptic gastroscopy biopsy identified only moderate chronic superficial gastritis, accompanied by inflammatory exudate and necrotic tissue (Fig. 3). Following cervical lump resection, an abdominal laparotomy was performed and a 5.6×5.3×4.0-cm mass was found located in the posterior wall of the stomach. The mass was prominent in the gastric wall, with a clear border. The primary diagnosis was of a GIST due to the mesenchymal origin. The surgery was successful and specimens were sent for pathological examination. Microscopically, the excised tumor tissue was composed of spindle-shaped and fascicularly-arranged cells, but mitotic figures were rare (Fig. 4). Immunohistochemical staining showed that the tumor was negative for cluster of differentiation (CD)117, CD34, smooth muscle actin (SMA) and desmin, but positive for S-100 and Ki67 (<1%) (Fig. 5). As a result, the pathological diagnosis was of a cellular schwannoma. Since no post-operative complications occurred, the patient was treated and discharged 10 days after surgery. No evidence of recurrence was identified during 12 months of follow-up by ultrasonography or CT.

## Discussion

Cellular schwannoma, first reported by Woodruff *et al* in 1981 (1), is a rare pseudosarcoma (2) that is mainly found in middle-aged individuals, with no significant difference in incidence between males and females (3). The predilection is for the paraspinal region, particularly the mediastinal, retroperitoneal, pelvic and sacral areas, followed by the neck and limbs (4–6). Gastric cellular schwannoma is a rare mesenchymal tumor that arises from the nerve plexus of the gut wall. Clinically, its main manifestations are gastrointestinal bleeding, chronic abdominal pain and an abdominal mass, but it is often detected incidentally. The majority of cases show a solitary painless mass and are occasionally multifocal (7). Pre-operative investigation is not pathognomonic, as a number of cases are diagnosed as GISTs (8). Imaging studies, such as barium meal, ultrasound and CT, are used only for diagnosing the position of gastric cellular schwannoma, but the qualitative diagnosis is often extremely difficult. The use of magnetic resonance imaging is justified, since the tumor is hypointense on T1 scans; however, the hypointensity is more evident on T2 scans (9). The rate of omissions and incorrect diagnoses by gastrofiberscope is higher, mainly affected by the level of skill of the gastroscopy physician, the location and depth of the biopsy, and other factors. However, biopsy extraction may improve the diagnostic rate. Adopting the intraoperative frozen section procedure is feasible if the conditions are permissible. The majority of cases are confirmed by pathology.

Gastric cellular schwannomas are easily misdiagnosed as various types of sarcoma in pathology, which leads to unnecessarily excessive treatments. We believe that the following criteria may aid the diagnosis of cellular schwannomas. In general, a cellular schwannoma is circular or elliptical in shape, with a diameter between 1–23 cm and an average diameter of 5.2 cm. The incisional surface is greyish to sallow. In addition, a mosaic-like distribution of yellow nodules is visible in specific cases, with focal bleeding, but no cystic changes. Histologically, a cellular schwannoma exhibits thick fibrous capsules, gathered subcapsular or extracapsular lymphocytes, visible foam cells and hyaline degeneration of the thick-walled blood vessels within the tumor. The tumor cells are spindle-shaped, with a fascicular or interfelted arrangement (Antoni A) and high cell density, lacking the classic schwannoma reticular zone (Antoni B) and palisade arrangement structures (Verocay bodies). The vortex-like structure that is used as an indicator of neuroal differentiation was occasionally visible. In specific cases, the tumor cells display a certain degree of pleomorphism and a small number of mitotic figures (<4/10 HPF), but do not exhibit pathological mitotic figures or coagulation necrosis. Immunohistochemical staining is of great value in the differential diagnosis of this tumor (Table II) (10); the tumor is negative for S-100, glial fibrillary acidic protein (GFAP) and CD57 in the cellular schwannoma, and positive for CK (AE1/AE3), desmin, SMA, CD34, CD117 and discovered on GIST1 (DOG1), with a low positive rate for Ki67.

According to the pathological characteristics, gastric cellular schwannomas are primarily identified with the following tumors: A GIST is the most common gastrointestinal mesenchymal tumor, with clinical imageology characteristics extremely close to that of gastric cellular schwannoma (11). The gastric cellular schwannoma cells exhibit a short spindle or a spindle similar to that of GISTs with a predominance of spindle cells, generally intertwined in a short article bundle or swirling arrangement. Therefore, gastric cellular schwannoma is occasionally difficult to identify. Immunohistochemically, gastric cellular schwannoma has been found to be positive for CD117, CD34 and DOG1 and negative for GFAP. Previously, it has been reported that gastric cellular schwannoma may be contaminated with small focal GISTs (12), therefore, a biopsy from numerous positions is necessary. Gastric myogenic tumors are rare. The sections of leiomyomas and leiomyosarcomas are often gray, exhibit braided structures, red cytoplasm, rod- and cigar-shaped visible nuclei and visible vacuoles. The tumor is positive for myogenic markers, not diffusely positive for S-100 and negative for GFAP. Solitary fibrous tumors (SFTs) are composed of rich and sparse areas of cells in alternating distributions. Among the tumor cells containing collagen, there are fibers of varying thickness and shape, with long spindle cells, but no Antoni A and B areas. However, a certain degree of CD34 and bcl-2 expression is likely to be evident. Gastric malignant peripheral nerve sheath tumors are extremely rare. Atypia of the tumor cells, necrosis and the common presence of mitotic figures may aid diagnosis. Gastric malignant peripheral nerve sheath tumors lack subcapsular or extracapsular lymphocyte sets, foamy histiocyte aggregates between the spindle cells, perivascular lymphocytic infiltration and vascular wall hyalinosis, which gastric cellular schwannomas display morphologically. The majority of gastric malignant peripheral nerve sheath tumors are associated with neurofibromatosis (13). However, certain studies have speculated that malignant peripheral nerve sheath tumors of the stomach are GISTs (14). Neurofibromas do not exhibit Antoni A and B areas when using microscopy. The immunophenotype of neurofibroma is often not diffusely positive for S-100 expression. A solitary neurofibroma of the stomach is extremely rare (15). Fibrosarcoma cells express only vimentin and are negative for S-100, GFAP and neurogenic markers. Gastrointestinal autonomic nerve tumors are more common in the small intestine, followed by the stomach, and are prone to being classified as a stromal tumor with neuronal differentiation (11). In addition to gastric cellular schwannomas, malignant fibrous histiocytomas, inflammatory myofibroblastomas and other types of tumor occasionally exhibit lymphoid hyperplasia and follicles at the tumor edge. However, their histological morphologies are extremely different compared with schwannoma, so are not difficult to identify.

Cellular schwannomas are not sensitive to radiotherapy or chemotherapy (16). Currently, a complete surgical resection of the tumor is the only effective method of treatment (17). The final diagnosis of gastric cellular schwannoma relies on the pathology and immunohistochemistry (18–19). The type of benign or malignant tumor is based mainly on the nuclear mitotic figures and heteromorphisms of the tumor cells. The majority of gastric cellular schwannomas are benign, with good prognosis and a low recurrence rate, but 10–15% of the tumors are malignant (20). According to the location, size and peculiarity of the tumor, the appropriate surgical procedure may be selected. Gastric cellular schwannomas rarely exhibit lymph node metastasis, so a regional lymph node dissection is not required. For the growth of the tumor in the gastric lumen, which is small, endoscopic removal may be performed or combined with laparoscopic surgery for the tumor resection. For the growth of the tumor outside of the gastric lumen, a laparoscopic removal, gastric wedge resection or subtotal gastrectomy may be performed. Minimally invasive surgery has the advantages of a rapid post-operative recovery and little trauma, and following the establishment of pneumoperitoneum, there is no requirement to consider the metastasis of tumor cells in the abdominal cavity. Therefore, minimally invasive surgery may be used as the preferred surgical treatment for gastric cellular schwannomas. For larger, suspected malignant tumors and in the minimally invasive treatment of a difficult situation, conventional open surgery is recommended. Radical gastrectomy may be performed on malignant tumors, but these often exhibit distant metastasis, resulting in a poor efficacy (21). The specific substances expressed in gastric cellular schwannoma must be identified to highlight a theoretical basis for molecular-targeted therapy (22). The patient in the current case exhibited no abdominal abnormalities, but a pre-operative routine examination for a neck mass identified the abdominal mass. Therefore, the patient was diagnosed and treated early. The patient did not exhibit any specific symptoms or signs. In addition, a radiological examination did not reveal any specific signs, as the clinical physician's diagnostic focus was too narrow and the examination was not comprehensive. Furthermore, the biopsy position of the gastrofiberscope was too superficial and the intraoperative biopsy did not undergo rapid freezing, resulting in a misdiagnosis. The performed radical resection resulted in the excessive treatment of the patient. The current report consequently provides a warning to clinicians when diagnosing cellular schwannoma.

## Figures and Tables

**Table I:** Laboratory observations upon admission.

Tumor markers	Index	Normal range
CA19-9, KU/l	8.96	<35.00
NSE, ng/ml	5.74	<13.00
CEA, ng/ml	2.03	<5.00
CA242, KU/l	5.47	<20.00
Ferritin, ng/ml	16.58	<322.00
β-HCG, MIU/ml	0.39	<3.00
AFP, ng/ml	0.32	<20.00
Free-PSA, ng/ml	0.06	<1.00
PSA, ng/ml	0.57	<5.00
CA125, KU/l	1.06	<35.00
HGH, ng/ml	1.18	<7.50
CA15-3, KU/l	3.07	<35.00

CA, cancer antigen; NSE, neuron-specific enolase; CEA, carcinoembryonic antigen; β-HCG, human chorionic gonadotropin-β; AFP, α fetoprotein; PSA, prostate-specific antigen; HGH, human growth hormone.

**Table II:** Immunohistochemical indices for the differential diagnosis of mesenchymal tumors.

Tumor	CD34	bcl-2	CD99	S-100	Cytokeratin	EMA	Calretinin	Desmin	SMA
Cellular schwannoma	±	+		+					
Neurofibroma	+	+		+					
Spindle cell lipoma	+	+							
Synovial sarcoma	−	+	±		+	+			
Desmoid tumor	−				−	−		+	+
Hemangiopericytoma	+	−	±					−	−
Malignant peripheral nerve sheath tumor	±	±		+	±			±	±
Sarcomatoid mesothelioma	−	±	±		+	+	+		
SFT	+	+	+		−	−	−	±	±
Calcifying fibrous pseudotumor	±				−		−	−	
Smooth muscle tumor	±	±	±					+	+

EMA, epithelial membrane antigen; SMA, smooth muscle actin; SFT, solitary fibrous tumor; CD, cluster of differentiation.
